# Unsafe, Unscientific, Unreliable!

**Published:** 1899-03

**Authors:** 


### UNSAFE, UNSCIENTIFIC, UNRELIABLE!

That is the charge we make against Vaccine "points," and it will be borne out by every bacteriologist and every experienced vaccinator.

In the first place, an unnecessarily large proportion of "points" fail to "work," and this can not be explained away by the plea of "natural immunity." It is true that many individuals resist the action of vaccine and can not possibly be inoculated, but these "immunes" form a relatively small percentage, and to lay at their door the failures of an inert or inferior vaccine is a lame evasion. As Dr. Moran reports, our Glycerinated Vaccine yielded, out of 87 doses, 83 successful primary vaccinations!

"Points" are unsafe, because they are very frequently contaminated with germs. Why are so many "successful" vaccinations attended with swollen arms, fever, general malaise? Singly and solely as a result of septic infection. It is ridiculous to commend that a pure and *aseptic* vaccine must necessarily lay *any* man up in bed, unless it be administered in a bungling manner. The peculiar merit of our Vaccine is its activity *plus* its freedom from distressing side or after-effects.

Our Glycerinated Vaccine is "scientific." Its production is attended with as much painstaking care as prevails in the finest modern hospitals when dangerous operations are performed. An operation for appendicitis could hardly involve more prolonged and minute and repeated use of disinfectants and antiseptics than does our method of inoculating the calves and securing the lymph. Our Vaccine is strictly aseptic, and we keep it so by marketing it in hermetically sealed tubes.

Our Vaccine is physiologically tested—every single parcel! Hence we know and guarantee it to be active.

CAUTION: Do not pull down the sleeve or bandage the arm till the Vaccine has dried on the wound. This is important. Please note Dr. Moran's procedure.

Net to the trade, in cases containing ten capillary tubes and one rubber bulb for ejecting the lymph, 80 cents.

